# Supervised injection facility use and all-cause mortality among people who inject drugs in Vancouver, Canada: A cohort study

**DOI:** 10.1371/journal.pmed.1002964

**Published:** 2019-11-26

**Authors:** Mary Clare Kennedy, Kanna Hayashi, M-J Milloy, Evan Wood, Thomas Kerr

**Affiliations:** 1 British Columbia Centre on Substance Use, St. Paul’s Hospital, Vancouver, British Columbia, Canada; 2 Department of Medicine, University of British Columbia, St. Paul’s Hospital, Vancouver, British Columbia, Canada; 3 Faculty of Health Sciences, Simon Fraser University, Burnaby, British Columbia, Canada; Massachusetts General Hospital, UNITED STATES

## Abstract

**Background:**

People who inject drugs (PWID) experience elevated rates of premature mortality. Although previous studies have demonstrated the role of supervised injection facilities (SIFs) in reducing various harms associated with injection drug use, including accidental overdose death, the possible impact of SIF use on all-cause mortality is unknown. Therefore, we examined the relationship between frequent SIF use and all-cause mortality among PWID in Vancouver, Canada.

**Methods and findings:**

Data were derived from 2 prospective cohort studies of PWID in Vancouver, Canada, between December 2006 and June 2017. Every 6 months, participants completed questionnaires that elicited information regarding sociodemographic characteristics, substance use patterns, social-structural exposures, and use of health services including SIFs. These data were confidentially linked to the provincial vital statistics database to ascertain mortality rates and causes of death. We used multivariable extended Cox regression analyses to estimate the independent association between frequent (i.e., at least weekly) SIF use and all-cause mortality. Of 811 participants, 278 (34.3%) were women, and the median age was 39 years (IQR 33–46) at baseline. In total, 432 (53.3%) participants reported frequent SIF use at baseline, and 379 (46.7%) did not. At baseline, frequent SIF users were on average younger than nonfrequent users, and a higher proportion of frequent SIF users than nonfrequent users were unstably housed, resided in the Downtown Eastside neighbourhood, injected in public, had a recent non-fatal overdose, used prescription opioids at least daily, injected heroin at least daily, injected cocaine at least daily, and injected crystal methamphetamine at least daily. A lower proportion of frequent SIF users than nonfrequent users were HIV positive and enrolled in addiction treatment at baseline. The median duration of follow-up among study participants was 72 months (IQR 24–123). In total, 112 participants (13.8%) died during the study period, yielding a crude mortality rate of 22.7 (95% CI 18.7–27.4) deaths per 1,000 person-years. The median years of potential life lost per death was 34 (IQR 27–42) years. In a time-updated multivariable model, frequent SIF use was inversely associated with risk of all-cause mortality after adjusting for potential confounders, including age, sex, HIV seropositivity, unstable housing, at least daily cocaine injection, public injection, incarceration, enrolment in addiction treatment, and calendar year of interview (adjusted hazard ratio 0.46, 95% CI 0.26–0.80, *p* = 0.006). The main study limitations are the limited generalizability of findings due to non-random sampling, the potential for reporting biases due to reliance on some self-reported information, and the possibility that residual confounding influenced findings.

**Conclusions:**

We observed a high burden of premature mortality among a community-recruited cohort of PWID. Frequent SIF use was associated with a lower risk of death, independent of relevant confounders. These findings support efforts to enhance access to SIFs as a strategy to reduce mortality among PWID. Further analyses of individual-level data are needed to determine estimates of, and potential causal pathways underlying, associations between SIF use and specific causes of death.

## Abstract 2

Thomas Kerr and co-authors investigate all-cause mortality in people who frequently used a supervised injection facility to inject drugs.

## Author summary

**Why was this study done?:**

- Previous studies have indicated that supervised injection facilities contribute to reductions in overdose-related deaths. However, it is not known if supervised injection facility use may shape risk of all-cause mortality.
- From a public health perspective, this is an important topic to investigate given the urgent need for evidence-based interventions to address the disproportionately high rates of premature mortality experienced by people who inject drugs in many settings internationally.

**What did the researchers do and find?:**

- In this study, we prospectively followed a community-recruited cohort of 811 people who inject drugs in Vancouver, Canada, for a median follow-up duration of 6 years.
- We longitudinally assessed the association between frequent supervised injection facility use and all-cause mortality using extended Cox regression with time-updated covariates.
- We found that this cohort of people who inject drugs experienced a high burden of premature mortality. A total of 112 participants (13.8%) died during follow-up, yielding a crude mortality rate of 22.7 (95% confidence interval 18.7–27.4) deaths per 1,000 person-years and a median of 34 years of potential life lost (interquartile range 27–42) per death.
- We also found that individuals who reported using supervised injection facilities on an at least weekly basis had a reduced risk of dying compared to those who reported less than weekly or no use of this health service. This association held after statistical adjustment for potential confounders including age, sex, HIV seropositivity, unstable housing, at least daily cocaine injection, public injection, incarceration, enrolment in addiction treatment, and calendar year of interview (adjusted hazard ratio 0.46, 95% confidence interval 0.26–0.80, *p* = 0.006).

**What do these findings mean?:**

- These findings suggest that increasing access to supervised injection facilities may help to prevent premature mortality among people who inject drugs.
- Additional studies should be conducted to determine individual-level estimates of the impact of supervised injection facility use on specific causes of death, and to discern possible underlying mechanisms that may account for these potential associations.

## Introduction

People who inject drugs (PWID) are known to be at heightened risk of premature mortality. A 2013 systematic review and meta-analysis of 67 cohort studies estimated that PWID worldwide have a crude all-cause mortality rate of 2.4 deaths per 100 person-years, a rate 14.7 times that of the general population [1]. Globally, the leading causes of death among PWID are accidental drug overdose and HIV-related disease [1], and in the US and Canada in particular, overdose deaths have increased dramatically in recent years to become a leading cause of accidental death at the general population level [2,3]. As a result of this rise in overdose deaths, average life expectancy of the general population has recently declined in the US, and has failed to increase in Canada for the first time in over 4 decades [3,4]. In addition, previous studies undertaken in diverse settings internationally have found that other underlying causes of death, including suicide, liver-related conditions, and other non-accidental causes (e.g., circulatory and respiratory infections or diseases), are also common among PWID [5–9].

As part of efforts to address the health and social harms stemming from injection drug use, including mortality and morbidity related to overdose and infectious diseases, an increasing number of cities worldwide have opened supervised injection facilities (SIFs) [10,11]. SIFs provide regulated spaces in which individuals can inject previously acquired illicit drugs under the supervision of health professionals or trained staff [11]. Within SIFs, clients are typically provided with sterile drug use equipment, education on safer drug consumption practices, emergency intervention in the event of overdose, and referrals to co-located and external addiction treatment and health services [11]. At present, more than 140 SIFs are in operation internationally, including in Canada, Australia, and Europe [10–15].

In 2003, North America’s first government-sanctioned SIF, Insite, was established in the Downtown Eastside of Vancouver, Canada, a neighbourhood characterized by a large open drug scene and high levels of marginalization and criminalization [16]. This facility remained the only sanctioned SIF in North America until 2016, when additional SIFs began to be established and legally authorized in Canada in response to the overdose crisis [16]. Since then, a total of 39 SIFs have been federally sanctioned and are now operating in cities across the country, 3 of which are located in Vancouver [12]. In addition, more than 30 provincially sanctioned low-threshold SIFs, known as overdose prevention sites, have been implemented in Canada since 2016, 6 of which are presently operating in Vancouver [13,14,16,17]. In the US, no SIFs have received formal legal sanctions to operate to date, although several major cities are currently considering authorizing such facilities, and an unsanctioned SIF has been operating in an undisclosed urban area in the country since 2014 [18].

Evaluations of SIFs in Canada and international settings have provided extensive evidence of the effectiveness of this form of health intervention [11,19,20]. For instance, past studies have consistently shown that SIFs effectively attract and retain their target client population, including PWID who contend with structural vulnerabilities (e.g., homelessness) and engage in drug use practices associated with heightened risk of morbidity and mortality (e.g., public injection, binge injection, frequent injection) [6,21–31]. Additionally, studies have identified associations between SIF use and various positive changes in health-related outcomes among PWID, including reduced likelihood of engaging in injection practices associated with infectious disease transmission (e.g., syringe sharing), as well as increased uptake of addiction treatment and other health and social services [21,32–43]. Past research has also found that SIFs contribute to reductions in overdose-related morbidity and mortality [13,44–49]. For example, a geospatial analysis of death records demonstrated that the establishment of Insite in Vancouver was associated with a 35% population-level decrease in the fatal overdose rate in the area surrounding the SIF, compared to a 9% decrease in the rest of the city [44]. Further, a recent mathematical modelling study estimated that between 160 and 350 overdose deaths were averted by SIFs operating in Vancouver and other municipalities in British Columbia between April 2016 and December 2017 [13].

Although these latter analyses indicate a protective role of SIFs against overdose mortality, we know of no studies that have examined the potential impact of SIF use on all-cause mortality. Information concerning the relationship between SIF use and mortality may be of public health importance given that evidence-based interventions to mitigate premature death among PWID are urgently needed at present, and that many jurisdictions in Canada and elsewhere are currently debating the merits of implementing SIFs as a strategy to address drug-related harms [12,16,18]. We therefore undertook the present study to examine the association between frequent SIF use and all-cause mortality among a community-recruited cohort of PWID in Vancouver, Canada, between 2006 and 2017. We also sought to examine the frequency and distribution of premature mortality in this cohort by estimating the years of potential life lost (YPLL) among individuals who died during follow-up.

## Methods

### Study sample

The Vancouver Injection Drug Users Study (VIDUS) and the AIDS Care Cohort to evaluate Exposure to Survival Services (ACCESS) are 2 concurrent community-recruited prospective cohort studies of people who use drugs in Vancouver, Canada. Participants have been recruited through self-referral, snowball sampling, and street outreach since May 1996. These cohorts have been described in detail previously [50,51]. In brief, persons are eligible for VIDUS if they report having injected illicit drugs at least once in the previous month at enrolment. Persons are eligible for ACCESS if they are HIV-infected and report having used illicit drugs in the previous month at enrolment. Individuals who seroconvert following recruitment are transferred from VIDUS into ACCESS, although ACCESS also includes individuals not previously followed in VIDUS who meet the ACCESS study eligibility criteria. All enrolled study participants provide written informed consent. The VIDUS and ACCESS studies have been approved by the University of British Columbia/Providence Health Care Research Ethics Board (H05-50234; H05-50233; H14-01396).

At baseline and every 6 months thereafter, study participants in both cohorts complete a harmonized interviewer-administered questionnaire that elicits information regarding sociodemographic characteristics, drug use and other behavioural patterns, social-structural exposures, and use of health services including SIFs. In addition, participants provide blood samples for HIV testing or disease monitoring, as appropriate, and hepatitis C testing. At the conclusion of each study visit, participants receive a Can$40 honorarium.

We restricted the present analyses to participants who completed at least 1 baseline or follow-up interview between December 1, 2006, and June 30, 2017 (the time period during which all variables of interest were available) in which they reported having injected drugs in the previous 6 months. As previously mentioned, SIF use has been associated with a number of notable health benefits for PWID [11,19,20]. However, existing literature also indicates that PWID who engage with this health service tend to be more likely than non-users to possess various markers of structural vulnerability and drug-related risk and therefore may have an inherently greater risk of death [6,21–31]. We expected that such selection effects would preclude individuals who had never used SIFs from being an appropriate comparison population when examining the association between frequent SIF use and mortality, as has been described in studies of frequent needle exchange use [52]. Thus, in effort to mitigate potential bias due to lack of comparability of exposure variable groups (with respect to balance of potential confounding factors) when estimating this association [53–56], we further restricted our analyses to participants who reported having used a SIF use at least once in the past 6 months in ≥50% of their available study visits. The ≥50% of available study visits cutoff point was employed for this restriction criterion given that participants who reported having used a SIF at least once during follow-up reported past-6-month SIF use in a median of 53.8% of their available study visits. Thus, applying this sample restriction was intended to exclude individuals who rarely or never used SIFs during follow-up and who therefore may have systematically differed in terms of their overall mortality risk profile in comparison to those who used this health service more consistently during follow-up. We expected that this approach would allow us to minimize the potential for bias due to selection effects and confounding when estimating the association of interest by reducing variation in the values of confounders, including unknown and unmeasured confounders, in the study sample [53,55,56].

### Measures

The primary outcome for this analysis was all-cause mortality. This variable and specific underlying causes of death were ascertained through confidential record linkages with the British Columbia Vital Statistics Agency, the centralized mortality registry for the province, using government-issued personal health numbers. The Vital Statistics Agency database recorded causes of death during the study period in accordance with the International Classification of Diseases and Related Health Problems–10th Revision (ICD-10) codes used in medical records. To avoid potential bias due to long durations between study visits and death [6], individuals who died more than 24 months after their last recorded follow-up visit were censored on the date of their last study visit. Consistent with previous studies of PWID [1,6–8], causes of death were classified into the following 8 categories: HIV-related, overdose, liver-related, homicide, suicide, other accidental, other non-accidental, and ill-defined/unknown causes. The primary exposure of interest was frequent SIF use. This was defined in response to one of the following questions: “In the last 6 months, how often have you used Insite to inject?” (December 2006 to November 2016) or “In the last 6 months, how often have you used supervised injection facilities to inject?” (December 2016 to June 2017, as additional SIFs and overdose prevention sites began operating in Vancouver in December 2016 [16]). Consistent with our past work [33,35], responses were classified as at least once a week versus less than once a week (including no use).

To examine the independent association between frequent SIF use and all-cause mortality, we assessed the following as potential confounding variables on the basis of previous literature concerning mortality and SIF use among PWID [1,6,8,21–26,29]: age (per year older), sex (male versus female), ancestry (white versus non-white), HIV status (positive versus negative serological test); hepatitis C virus status (positive versus negative serological test), and heavy alcohol use (average of >3 alcoholic drinks per occasion at least once per week or >7 drinks in total per week in the previous 6 months for women, and average of >4 alcoholic drinks per occasion at least once per week or >14 drinks in total per week in the previous 6 months for men [57]). Other potential confounders examined included Downtown Eastside residence, unstable housing, binge injection drug use, public injection drug use, non-fatal overdose, enrolment in addiction treatment, exposure to violence, incarceration, involvement in sex work, and benzodiazepine use (all yes versus no). Finally, we assessed as confounders frequent use of injection heroin, injection cocaine, injection crystal methamphetamine, non-injection crack cocaine, injection or non-injection prescription opioids, and cannabis (all at least daily versus less than daily). Variable definitions were consistent with those used in our previous work [6,35,58,59]. Unless otherwise indicated, all variables refer to activities and experiences that occurred in the 6-month period preceding the date of the interview, and were treated as time-updated based on each semi-annual follow-up visit.

### Analysis

First, we examined descriptive statistics and estimated odds ratios to compare the baseline characteristics of cohort participants who were included in the study with those who were not. Next, we calculated the crude mortality rates and 95% confidence intervals [CIs] for all-cause mortality and each specific cause of death using the Poisson distribution. To investigate premature mortality among the study sample, we calculated the YPLL for each decedent using the method described by Aragón and colleagues [60]. As previously [61,62], we used conservative life expectancy estimates based on data for the province of British Columbia from Statistics Canada (84.6 years for females and 80.1 years for males) [4] and calculated the median YPLL per death and rate of YPLL per 100,000 population. We then examined descriptive statistics and estimated odds ratios to compare baseline characteristics of those who reported frequent SIF use at baseline with those who did not. Next, we used bivariable extended Cox regression analyses with time-updated covariates to examine the association between each explanatory variable (i.e., frequent SIF use and all hypothesized potential confounders) and all-cause mortality. We then applied an a priori–defined statistical protocol to estimate the independent association between frequent SIF use and all-cause mortality. First, we fit a multivariable model that included frequent SIF use and all hypothesized potential confounders as explanatory variables. Next, we removed the hypothesized confounding variable corresponding to the smallest relative change in the frequent SIF use coefficient. We continued this iterative process until the minimum change in the value of the coefficient for frequent SIF use exceeded 5%. Lastly, age, sex, and unstable housing were forced into the model to account for the established associations between these variables and the primary exposure and outcome variables of interest [6,21–26,29,58]. For all participants, time 0 was defined as the date of first report of past-6-month injection drug use during the study period given that only active injectors are eligible to use SIFs. Participants who did not die during follow-up were right censored at the date of their latest interview, their first report of having not injected drugs in the previous 6 months, or June 30, 2017, whichever came first. We also conducted sensitivity analyses to determine whether using an alternative measure of SIF use or broadening our study sample inclusion criteria would significantly alter our results (see S1 Text). We conducted all statistical analyses with SAS version 9.4 (SAS Institute, Cary, NC), and all reported *p*-values are 2-sided. The study analysis plan is included as S2 Text. The study is reported in accordance with the Strengthening the Reporting of Observational Studies in Epidemiology (STROBE) guidelines for cohort studies (see S1 STROBE Checklist).

## Results

**Fig 1:**
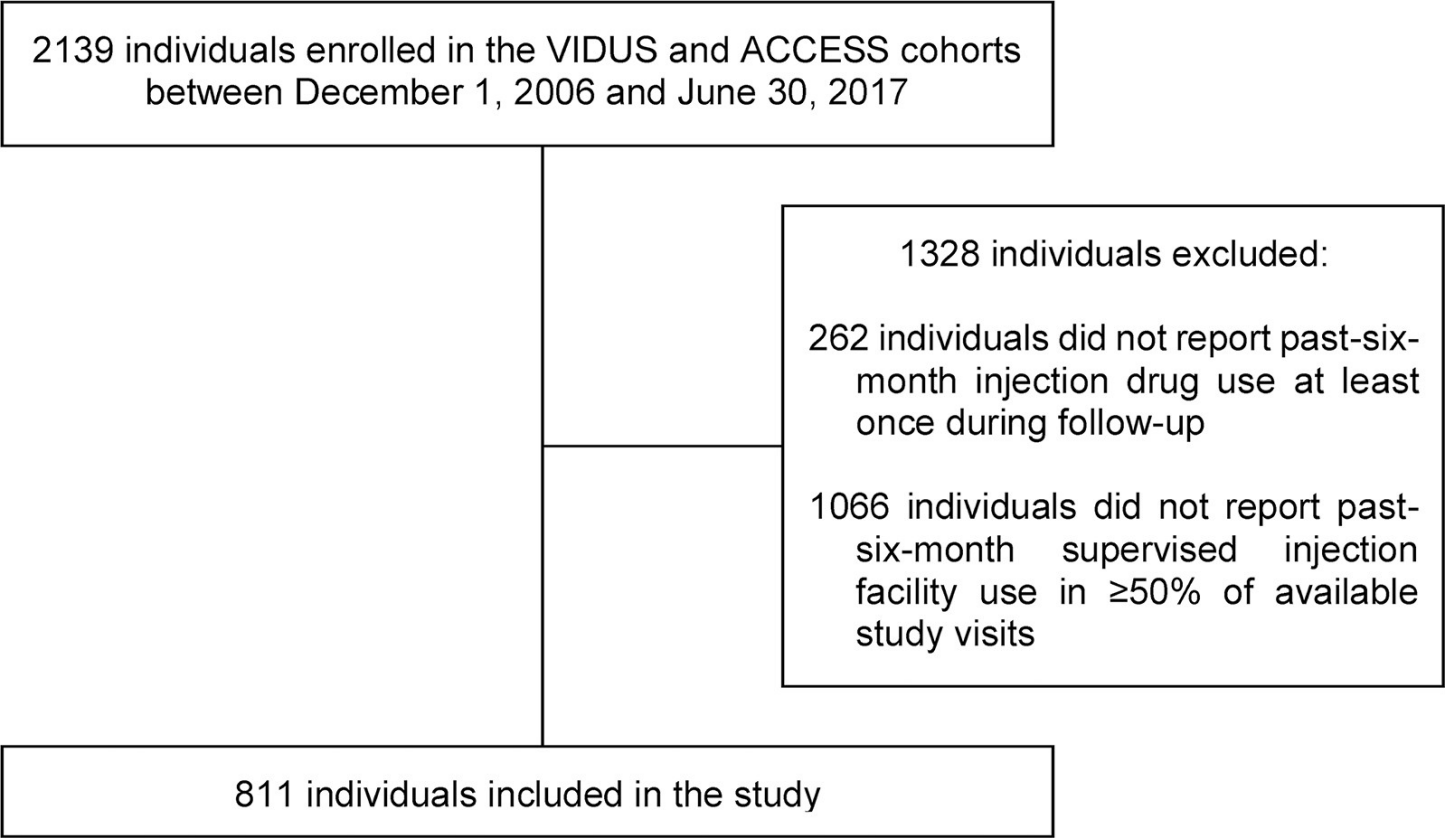
Flowchart showing how the analytical sample (*n* = 811) was determined. ACCESS, AIDS Care Cohort to evaluate Exposure to Survival Services; VIDUS, Vancouver Injection Drug Users Study.

Between December 2006 and June 2017, 2,139 participants were recruited into the cohorts. As shown in Fig 1, 1,328 individuals were excluded from the present study because they either did not report past-6-month injection drug use in any study interviews during the study period (*n* = 262) or did not report past-6-month SIF use in at least 50% of their available interviews (*n* = 1,066). Compared with participants included in the analytic sample (*n* = 811), those excluded (*n* = 1,328) were more likely to be older, be HIV seropositive, and report heavy alcohol use at baseline (all *p* < 0.05). Additionally, participants excluded from the analytic sample were less likely than those included to reside in the Downtown Eastside, be unstably housed, be hepatitis C seropositive, inject heroin at least daily, inject cocaine at least daily, inject crystal methamphetamine at least daily, use prescription opioids at least daily, use crack cocaine at least daily, inject in public, binge inject, have had a recent non-fatal overdose, have recently experienced violence, have recently engaged in sex work, and have been recently incarcerated at baseline (all *p* < 0.05). S3 Text reports the results of analyses comparing the baseline characteristics of individuals who reported past-6-month SIF use in at least 50% of their available study visits and were therefore included in the analytic sample (*n* = 811) versus those who did not and were therefore excluded from the analytic sample (*n* = 1,066) among cohort participants who completed at least 1 interview during the study period in which they reported having injected drugs in the previous 6 months (*n* = 1,877).

The 811 PWID included in present study were followed for a median duration of 72 months (interquartile range [IQR] 24–123) and collectively contributed a total of 4,928.1 person-years of observation. At baseline, 278 (34.3%) study participants were women, and the median age was 39 years (IQR 33–46). A total of 432 (53.3%) participants reported frequent (i.e., at least weekly) SIF use at baseline. Table 1 reports the baseline characteristics of the study participants stratified by frequent SIF use. As shown, at baseline, persons who reported frequent SIF use were more likely than those who did not to be younger (median age = 38 versus 40 years), reside in the Downtown Eastside (84.7% versus 75.9%), be unstably housed (85.4% versus 78.1%), inject heroin at least daily (52.9% versus 30.1%), inject cocaine at least daily (17.4% versus 6.3%), inject crystal methamphetamine at least daily (12.8% versus 7.4%), use prescription opioids at least daily (14.2% versus 5.8%), inject in public (64.4% versus 52.4%), have had a recent non-fatal overdose (14.0% versus 9.5%), and have been recently incarcerated (31.5% versus 16.6%). Those who reported frequent SIF use at baseline were less likely to be HIV seropositive (25.3% versus 36.2%) and to be enrolled in addiction treatment (48.8% versus 56.7%) at baseline.

**Table 1:** Characteristics of 811 people who inject drugs in Vancouver, Canada, stratified by at least weekly supervised injection facility (SIF) use at baseline, 2006–2017.

Characteristic	Total (*n* = 811)	At least weekly SIF use*		Odds ratio (95% CI)
		Yes (*n* = 432)	No (*n* = 379)	
**Age**				
Median [IQR]	39 [33–46]	38 [32–45]	40 [33–48]	0.98 (0.96–0.99)
**Sex**				
Male	532 (65.7)	281 (65.4)	250 (66.0)	0.97 (0.73–1.30)
Female	278 (34.3)	149 (34.7)	129 (34.0)	
**Ancestry**				
White	526 (64.9)	282 (65.6)	244 (64.4)	1.05 (0.79–1.41)
Non-white	284 (35.1)	148 (34.4)	135 (35.6)	
Downtown Eastside residence*				
Yes	653 (80.5)	366 (84.7)	287 (75.9)	1.76 (1.24–2.50)
No	158 (19.5)	66 (15.3)	91 (24.1)	
Unstable housing*				
Yes	663 (81.9)	367 (85.4)	296 (78.1)	1.63 (1.14–2.35)
No	147 (18.2)	63 (14.7)	83 (21.9)	
HIV seropositive*				
Yes	246 (30.3)	109 (25.3)	137 (36.2)	0.60 (0.44–0.81)
No	566 (69.7)	322 (74.7)	242 (63.9)	
Hepatitis C seropositive*				
Yes	691 (85.3)	375 (87.0)	315 (83.3)	1.34 (0.91–1.98)
No	119 (14.7)	56 (13.0)	63 (16.7)	
Heroin injection*				
At least daily	342 (42.2)	228 (52.9)	114 (30.1)	2.61 (1.95–3.49)
Less than daily	469 (57.8)	203 (47.1)	265 (69.9)	
Cocaine injection*				
At least daily	99 (12.2)	75 (17.4)	24 (6.3)	3.13 (1.93–5.06)
Less than daily	711 (87.8)	355 (82.6)	355 (93.7)	
Crystal methamphetamine injection*				
At least daily	83 (10.3)	55 (12.8)	28 (7.4)	1.84 (1.14–2.97)
Less than daily	726 (89.7)	374 (87.2)	351 (92.6)	
Non-injection crack cocaine use*				
At least daily	314 (38.8)	177 (41.1)	137 (36.2)	1.23 (0.92–1.63)
Less than daily	496 (61.2)	254 (58.9)	241 (63.8)	
Prescription opioid use*				
At least daily	83 (10.2)	61 (14.2)	22 (5.8)	2.68 (1.61–4.45)
Less than daily	728 (89.8)	370 (85.9)	357 (94.2)	
Cannabis use*				
At least daily	174 (21.5)	86 (20.0)	88 (23.3)	0.82 (0.59–1.15)
Less than daily	635 (78.5)	345 (80.1)	289 (76.7)	
Benzodiazepine use*				
Yes	28 (3.5)	12 (2.8)	15 (4.0)	0.70 (0.32–1.50)
No	783 (96.6)	419 (97.2)	364 (96.0)	
Heavy alcohol use*^†^				
Yes	96 (11.8)	51 (11.8)	45 (11.9)	0.99 (0.65–1.52)
No	715 (88.2)	381 (88.2)	333 (88.1)	
Public injection*				
Yes	476 (58.8)	277 (64.4)	198 (52.4)	1.65 (1.24–2.18)
No	333 (41.2)	153 (35.6)	180 (47.6)	
Binge injection*				
Yes	264 (32.6)	140 (32.4)	123 (32.7)	0.99 (0.73–1.33)
No	545 (67.4)	292 (67.6)	253 (67.3)	
Non-fatal overdose*				
Yes	96 (11.9)	60 (14.0)	36 (9.5)	1.55 (1.00–2.40)
No	714 (88.2)	370 (86.0)	343 (90.5)	
Enrolled in addiction treatment*				
Yes	426 (52.6)	210 (48.8)	215 (56.7)	0.73 (0.55–0.96)
No	384 (47.4)	220 (51.2)	164 (43.3)	
Exposure to violence*				
Yes	242 (30.1)	140 (32.8)	102 (27.0)	1.32 (0.97–1.79)
No	563 (69.9)	287 (67.2)	276 (73.0)	
Sex work involvement*				
Yes	148 (18.3)	78 (18.2)	70 (18.5)	0.98 (0.68–1.40)
No	659 (81.7)	351 (81.8)	308 (81.5)	
Incarceration*				
Yes	198 (24.5)	135 (31.5)	63 (16.6)	2.30 (1.64–3.23)
No	610 (75.5)	294 (68.5)	316 (83.4)	

Data are provided as *n* (percentage) unless otherwise indicated. Column counts may not necessarily sum to column totals due to missing baseline data, and column percentages may not necessarily sum to 100% due to rounding error.

*Refers to the 6-month period prior to the baseline study visit.

^†^Average of >3 alcoholic drinks on at least 1 day per week or >7 drinks in total per week for women, or >4 alcoholic drinks on at least 1 day per week or >14 drinks in total per week for men.

SIF, supervised injection facility.

A total of 112 participants (13.8%) died during the 10.5-year study period, corresponding to a crude mortality rate of 22.7 deaths (95% CI 18.7–27.4) per 1,000 person-years. The underlying causes of death are presented in Table 2. The leading observed causes of death were as follows: other non-accidental (*n* = 30; 26.8%), ill-defined/unknown causes (*n* = 27; 24.1%), overdose (*n* = 19; 16.7%), and HIV-related causes (*n* = 15; 13.4%). The median YPLL per death was 33.6 (IQR 26.9–41.7) years, and the estimated rate was 3,431,827 (95% CI 3,231,297–3,632,356) YPLL per 100,000 population.

**Table 2:** Causes of death in a study of 811 people who inject drugs in Vancouver, Canada, 2006–2017.

Cause of death	*n*	Percent	Rate*	95% CI
**All causes**	112	100.0	22.7	18.7–27.4
**HIV-related**	15	13.4	3.0	1.7–5.0
**Overdose**	19	17.0	3.9	2.3–6.0
**Liver-related**	11	9.8	2.2	1.1–4.0
**Suicide**	3	2.7	0.6	0.1–1.8
**Homicide**	2	1.8	0.4	0.1–1.5
**Other accidental**	5	4.5	1.0	0.3–2.4
Substance-related	4	3.6		
Other causes	1	0.9		
**Other non-accidental**	30	26.8	6.1	4.1–8.7
Neoplasms	10	8.9		
Circulatory disease	8	7.1		
Respiratory disease	6	5.4		
Other causes	6	5.4		
**Ill-defined or unknown**	27	24.1	5.5	3.6–8.0

*Per 1,000 person-years.

Table 3 presents the crude and adjusted hazard ratios (HRs) for the associations between the explanatory variables and all-cause mortality. In bivariable extended Cox regression analyses, frequent SIF use was significantly and inversely associated with all-cause mortality (HR 0.57, 95% CI 0.34–0.94, *p* = 0.029). In the final multivariable Cox regression model, frequent SIF use remained significantly associated with decreased risk of all-cause mortality after adjusting for age, sex, HIV seropositivity, unstable housing, at least daily cocaine injection, public injection, incarceration, enrolment in addiction treatment, and calendar year of interview (adjusted HR 0.46, 95% CI 0.26–0.80, *p* = 0.006).

**Table 3:** Unadjusted and adjusted Cox regression analyses of factors associated with all-cause mortality among people who inject drugs (*n* = 811) in Vancouver, Canada, 2006–2017.

Characteristic	Unadjusted		Adjusted	
	Hazard ratio (95% CI)	*p-*Value	Hazard ratio (95% CI)	*p-*Value
**Age**				
Per year older	1.04 (1.01–1.07)	0.006	1.05 (1.01–1.09)	0.012
**Sex**				
Male versus female	1.54 (0.88–2.68)	0.128	1.62 (0.89–2.96)	0.114
**Ancestry**				
White versus non-white	0.80 (0.49–1.28)	0.345		
Downtown Eastside residence*				
Yes versus no	1.07 (0.65–1.76)	0.788		
Unstable housing*				
Yes versus no	1.16 (0.65–2.08)	0.614	1.39 (0.79–2.42)	0.250
HIV seropositive*				
Yes versus no	3.23 (2.00–5.24)	<0.001	4.28 (2.63–6.96)	<0.001
Hepatitis C seropositive*				
Yes versus no	0.99 (0.40–2.45)	0.978		
At least weekly supervised injection facility use*				
Yes versus no	0.57 (0.34–0.94)	0.029	0.46 (0.26–0.80)	0.006
At least daily heroin injection*				
Yes versus no	0.58 (0.34–0.99)	0.045		
At least daily cocaine injection*				
Yes versus no	1.67 (0.90–3.08)	0.101	1.47 (0.78–2.76)	0.232
At least daily crystal methamphetamine injection*				
Yes versus no	0.69 (0.28–1.72)	0.431		
At least daily non-injection crack cocaine use*				
Yes versus no	1.32 (0.80–2.21)	0.289		
At least daily prescription opioid use*				
Yes versus no	0.71 (0.29–1.73)	0.446		
At least daily cannabis use*				
Yes versus no	1.26 (0.71–2.26)	0.429		
Benzodiazepine use*				
Yes versus no	0.64 (0.16–2.55)	0.527		
Heavy alcohol use*^†^				
Yes versus no	1.30 (0.66–2.57)	0.453		
Public injection*				
Yes versus no	0.79 (0.49–1.28)	0.341	1.48 (0.93–2.37)	0.100
Binge injection*				
Yes versus no	0.88 (0.54–1.43)	0.591		
Non-fatal overdose*				
Yes versus no	0.76 (0.33–1.75)	0.518		
Enrolled in addiction treatment*				
Yes versus no	0.63 (0.40–1.01)	0.632	0.66 (0.41–1.08)	0.102
Exposure to violence*				
Yes versus no	0.63 (0.31–1.32)	0.221		
Sex work involvement*				
Yes versus no	0.97 (0.46–2.05)	0.941		
Incarceration*				
Yes versus no	0.37 (0.14–1.02)	0.055	0.43 (0.18–1.04)	0.060
**Calendar year of interview**				
Per year increase	0.60 (0.48–0.76)	<0.001	0.52 (0.40–0.69)	<0.001

*Refers to the 6-month period prior to a study visit.

^†^Average of >3 alcoholic drinks on at least 1 day per week or >7 drinks in total per week for women, or >4 alcoholic drinks on at least 1 day per week or >14 drinks in total per week for men.

## Discussion

In this 10.5-year study of a community-recruited cohort of more than 800 PWID in Vancouver, Canada, we observed a high burden of premature death, with an estimated crude mortality rate of 22.7 deaths per 1,000 person-years and a median of 34 YPLL per death. The primary causes of death were other non-accidental, ill-defined or unknown factors, accidental overdose, and HIV-related causes. We found that frequent SIF use was associated with lower risk of all-cause mortality, independent of potential confounders including sociodemographic characteristics, unstable housing, HIV seropositivity, at least daily cocaine injection, public injection, incarceration, enrolment in addiction treatment, and calendar year of interview.

Existing modelling and simulation studies indicate that SIFs avert numerous overdose deaths per year [13,48,49]. Moreover, past research relying on aggregate data has demonstrated the role of SIFs in reducing local population-based rates of fatal overdose [44,47]. However, we believe that ours is the first study to identify an individual-level association between frequent SIF use and decreased risk of all-cause mortality among a community-recruited cohort of PWID.

There are likely multiple explanations for the protective association between frequent SIF use and death observed in the present study. For instance, SIF use has been associated with positive changes in various injecting practices, including declines in syringe sharing, syringe reuse, outdoor injecting, and rushed injecting, thereby reducing the risk of acquiring HIV and other common viral and bacterial infections that may contribute to premature mortality [21,32,43,63]. In addition, the provision of rapid, well-equipped emergency response in the event of overdose within SIFs (e.g., oxygen and naloxone administration) has served to prevent the occurrence of on-site overdose deaths [11,19]. Indeed, no overdose deaths have ever occurred within any SIF in operation in Canada or internationally to date [11,19]. Further, regular SIF use and contact with addiction counsellors within SIFs have been associated with increased engagement with addiction treatment, including residential treatment and opioid agonist therapy [33–36,39], which may help to prevent deaths related to ongoing high-risk drug use [6,35,64–66]. SIFs may also mitigate mortality related to diverse causes by enhancing connections to other internal and external health and social services [37,38,40–42,67–72]. For example, studies of SIF clients in Vancouver have found that SIF nurses facilitate early intervention for the treatment of cutaneous injection-related infections, including by providing care for these conditions and referrals to hospital, which may prevent these from advancing to more severe forms of infection that could lead to death [37,38,70,71]. However, interpretations of the underlying explanations for the observed association between frequent SIF use and reduced risk of all-cause mortality cannot be confirmed based on the present analyses, and further investigation of these issues is warranted. In particular, future studies should seek to determine individual-level estimates of the impact of SIF use on specific causes of death, and to discern any mediating factors underlying these potential associations. This is especially important given that almost a quarter of the deaths included in the present study were listed in the Vital Statistics Agency database as being due to ill-defined or unknown causes, and therefore important questions remain about the pathways and mechanisms that may explain the observed protective relationship between SIF use and mortality among PWID in this setting.

Together with the findings of previous research [13,44,47,48], our findings underscore the need for continued efforts to enhance access to SIFs as a strategy to reduce mortality among PWID. In particular, given that SIFs have limited geographic coverage and that PWID have been found to often encounter long wait times in accessing SIF services in this setting, the broader expansion of SIFs may serve to improve service accessibility and thereby reduce the potential for mortality and other harms among this population [13,16,73–75]. The recent scale-up of SIFs in Vancouver and other settings in Canada provides an opportunity for future research to further examine these issues, including the potential impacts of this expansion on service utilization patterns and related health and social outcomes among PWID. As well, further efforts should be undertaken to mitigate other barriers to engagement with SIFs. For example, increasing SIF operating hours may promote more frequent use of this service, and amending SIF regulations that have been shown to constrain access to SIFs (e.g., rules prohibiting the provision of manual assistance with injections within most federally sanctioned SIFs in Canada) may help to engage vulnerable and underserved populations of PWID [25,74,76,77].

Our findings also point to the need for further research to better understand how varying levels of supplementary services offered within SIFs may shape risk of mortality among PWID. For example, studies should seek to determine if the association between service use and mortality differs between users of overdose prevention sites and users of conventional SIFs given that overdose prevention sites typically offer a lower level of ancillary services and supports (e.g., referrals, clinical care) [16]. Additionally, studies should continue to examine if specific programming co-delivered with SIF services (e.g., naloxone distribution programs, safer drug supply interventions, drug checking services, initiatives to support linkages to HIV care) may extend the health impacts of this intervention [68,69,78–80].

We should note that our sensitivity analyses involving an alternative 3-level measure of SIF use suggested an independent protective association between at least biweekly to less than daily SIF use (versus no SIF use to once monthly SIF use) and all-cause mortality, but did not suggest a significant association between at least daily SIF use (versus no SIF use to once monthly SIF use) and mortality (see S1 Text). While the latter finding may seem counterintuitive given the main findings of the present study, this finding likely reflects the extremely high-risk profile of daily SIF attendees [25], which may mask the protective benefits of SIF use when comparing these individuals to PWID who rarely or never use this health service, as has been found in studies of needle exchange use [52]. Although we sought to control for a range of potential confounders through sample restriction and statistical adjustment, and this shifted estimates of the association between at least daily SIF use and mortality in the direction of a protective association, there is significant potential for residual confounding due to failure to measure or imprecise measurement of notable potential confounders (e.g., socioeconomic marginalization) given the observational nature of this study, which may explain why this association did not achieve statistical significance.

This study has a number of additional limitations. Of note, the VIDUS and ACCESS cohorts are community-recruited, non-randomized samples of PWID, and therefore our findings may not be generalizable to PWID in Vancouver or other settings. Moreover, the main analyses presented in this study were restricted to PWID in the cohorts who reported recent SIF use in at least half of their available study visits, which likely further reduced the generalizability of our findings and decreased the precision of estimates of association. However, consistent with existing research [6,21–29,31], our findings indicate that many established risk factors for mortality were more prevalent among this group compared to individuals who were excluded from the study sample because they rarely or never used SIFs during the study period (see S3 Text). As such, we believe that our approach of restricting our analyses to this sample provided a more appropriate comparison population when examining the relationship between frequent SIF use and mortality by promoting balance across exposure variable groups with respect to known, unknown, and unmeasured confounders, thereby enhancing the internal validity of the study by reducing the potential for biased measures of association [53,55,56]. We should also note that although the main study sample was restricted to individuals who had used SIFs, we included observations in our analyses that captured heterogeneity in service use over time among these individuals, including periods in which SIFs were not used. In light of these strengths, future studies should continue to explore the application of this approach when evaluating potential impacts of SIF use. Another limitation is that this study relied on self-reported information for many measures, including SIF use given that service use was not recorded in administrative databases at some SIFs during the study period. Thus, our findings are susceptible to reporting biases, including social desirability bias. However, it is noteworthy that our primary outcome of mortality was based on objective measures derived from linkages to an external administrative database. As previously noted, a further limitation is that just under a quarter of all deaths observed in the present study were listed in the Vital Statistics Agency database as being due to ill-defined or unknown causes, which complicates interpretations of the observed protective association between SIF use and mortality. The observed excess of deaths of unknown causes is likely largely explained by delays in updating causes of death in the database in recent years as a result of a backlog in post-death toxicology testing due to the present overdose crisis [81]. Indeed, 55.6% of deaths of ill-defined or unknown causes observed in the present study occurred in the last 3 years of the study period. As such, the true prevalence of overdose-related deaths may have been underestimated in the present study, as may have been deaths of other specific causes. However, given that our primary study aim was to examine the independent association between SIF use and all-cause mortality (rather than distinct causes of death), we believe that the improvements in statistical power resulting from including recent deaths in our analyses offset the potential benefits concerning interpretations if we had instead restricted the study period to reduce the number of deaths of unknown causes. As mentioned previously, an additional limitation is that the observed relationship between frequent SIF use and decreased risk of mortality might be influenced by residual confounding. Although we sought to reduce the potential for this bias by restricting our study sample based on SIF utilization patterns and by adjusting multivariable analyses for key confounding factors, an e-value analysis [82] indicated that an unmeasured confounder associated with frequent SIF use and mortality by a HR equivalent to a magnitude of at least 1.81 each could explain away the upper confidence limit (i.e., the limit closest to the null value) for the observed adjusted HR for the association between frequent SIF use and all-cause mortality. For example, it is possible that we did not adequately adjust for social challenges associated with mortality risk that may be less prevalent among frequent SIF users compared to nonfrequent users, which could have biased our estimate of the association of interest away from the null. In particular, past qualitative research has documented how factors such as drug debts, street-level policing, and area restrictions (i.e., court-ordered restrictions prohibiting individuals from entering areas where they have been arrested) may deter some PWID from accessing health services concentrated within the local drug scene, including SIFs, and increase their susceptibility to harms [83–85]. However, as discussed above, existing evidence indicates that frequent SIF attendees are a particularly marginalized subpopulation of PWID who tend to be more likely than nonfrequent attendees to contend with various characteristics, behaviours, and exposures associated with heightened mortality risk [22,25]. As we likely imprecisely measured or neglected to measure some of such risk factors (e.g., markers of structural vulnerability and drug-related risk, comorbid conditions), we suspect that it is more probable that our observed estimate of the association between frequent SIF use and mortality is biased towards rather than away from the null.

In conclusion, this study of a cohort of PWID in Vancouver, Canada, reports a previously unidentified independent association between frequent SIF use and decreased risk of all-cause mortality. This relationship warrants further investigation. In particular, future studies should seek to examine the individual-level association between SIF use and distinct causes of death among PWID. Nonetheless, the findings of the present study suggest that efforts to scale up access to SIFs may serve to reduce preventable deaths among this population.

## Supporting information

S1 STROBE Checklist STROBE checklist. (DOCX)

S1 Text Sensitivity analyses. ACCESS, AIDS Care Cohort to evaluate Exposure to Survival Services; SIF, supervised injection facility; VIDUS, Vancouver Injection Drug Users Study.
(DOCX)

S2 Text Analysis plan. ACCESS, AIDS Care Cohort to evaluate Exposure to Survival Services; VIDUS, Vancouver Injection Drug Users Study.
(DOCX)

S3 Text Baseline characteristics of participants included versus excluded from the analytic sample on the basis of SIF use. ACCESS, AIDS Care Cohort to evaluate Exposure to Survival Services; PWID, people who inject drugs; SIF, supervised injection facility; VIDUS, Vancouver Injection Drug Users Study.
(DOCX)

## Abbreviations

ACCESS: AIDS Care Cohort to evaluate Exposure to Survival Services
PWID: people who inject drugs
SIF: supervised injection facility
VIDUS: Vancouver Injection Drug Users Study
YPLL: years of potential life lost
