# Supplementary material for: Supervised injection facility use and all-cause mortality among people who inject drugs in Vancouver, Canada: A cohort study
Source: PLoS Med. 2019 Nov 26;16(11):e1002964. doi: 10.1371/journal.pmed.1002964 (PMC6879115; doi:10.1371/journal.pmed.1002964)
Supplement: S1 Text — ACCESS, AIDS Care Cohort to evaluate Exposure to Survival Services; SIF, supervised injection facility; VIDUS, Vancouver Injection Drug Users Study. (DOCX) [file pmed.1002964.s002.docx]

**S1 Text - Sensitivity Analyses**

**Methods**

We recognized that there could be heterogeneity in the estimated measure of association between SIF use and all-cause mortality across different levels of SIF use beyond the two levels (≥weekly vs. weekly) examined in our main analysis. Thus, we replicated the extended Cox regression analyses described in the manuscript but with an alternative measure of SIF use as the primary explanatory variable of interest. This variable was defined in response to the same questionnaire items as the main binary measure of SIF use but with responses classified into the following three levels: ≥daily vs. every couple of weeks to every couple of days vs. no use to once a month. A multivariable model was built following the same approach as that used for the main analysis (see Analysis subsection of the Methods section in the manuscript).

We also sought to determine whether expanding our study sample inclusion criteria would substantially alter our estimates of the association between this three-level measure of SIF use and all-cause mortality. For this, the study sample was restricted to VIDUS and ACCESS participants who completed at least one study visit during the study period (December 1, 2006 and June 30, 2017) in which they reported having injected drugs in the previous six months. However, we did not further restrict to participants who reported past-six-month SIF use in ≥50% of available study visits (as we did in our main analysis). Bivariable and multivariable extended Cox regression analyses were conducted following the same approach used for the main analysis (see Analysis subsection of the Methods section).

**Results**

In bivariable analyses conducted among the main study sample (*n* = 811), individuals who reported having used a SIF every couple of weeks to every couple of days had a significantly lower hazard of dying compared to those who used SIFs once a month or less frequently (HR = 0.41; 95% CI = 0.23 – 0.76, *p* = 0.004). However, risk of all-cause mortality did not significantly differ between ≥daily SIF users and those who used SIFs once a month or less frequently in bivariable analyses (HR = 0.74; 95% CI = 0.39 – 1.41, *p* = 0.363). Table S1.1 presents the multivariable results for the association between the three-level measure of SIF use and all-cause mortality among the main study sample. As shown, ≥biweekly to <daily SIF use (compared to ≤once monthly or no SIF use) remained significantly associated with reduced risk of all-cause mortality after adjusting for age, sex, unstable housing, HIV seropositivity, public injection, enrolment in addiction treatment and calendar year of interview (HR = 0.35; 95% CI = 0.19 – 0.65, *p* <0.001). However, ≥daily SIF use (compared to ≤once monthly or no SIF use) was not significantly associated with all-cause mortality after adjusting for the same set of potential confounders (HR = 0.64; 95% CI = 0.33 – 1.23, *p* = 0.182).

| **Table S1.1 Adjusted Cox regression analyses of factors associated with all-cause mortality among supervised injection facility clients (*n* = 811) in Vancouver, Canada, 2006-2017.** | | | |
| --- | --- | --- | --- |
| **Variable** | **Adjusted  Hazard Ratio  (AHR)** | **95% Confidence Interval (CI)** | ***p -* value** |
| **Age** |  |  |  |
| (per year older) | 1.05 | (1.02 – 1.09) | 0.005 |
| **Sex** |  |  |  |
| (male vs. female) | 1.56 | (0.86 – 2.86) | 0.146 |
| **Unstable housing*** |  |  |  |
| (yes vs. no) | 1.28 | (0.72 – 2.25) | 0.402 |
| **HIV seropositive*** |  |  |  |
| (yes vs. no) | 4.41 | (2.72 – 7.14) | <0.001 |
| **Supervised injection facility use*** |  |  |  |
| (biweekly to every couple of days vs. ≤once a month) | 0.35 | (0.19 – 0.65) | <0.001 |
| (≥daily vs. ≤once a month) | 0.64 | (0.33 – 1.23) | 0.182 |
| **Public injection*** |  |  |  |
| (yes vs. no) | 1.39 | (0.87 – 2.22) | 0.167 |
| **Enrolled in addiction treatment*** |  |  |  |
| (yes vs. no) | 0.67 | (0.41 – 1.10) | 0.115 |
| **Calendar year of interview** |  |  |  |
| (per year increase) | 0.53 | (0.41 – 0.68) | <0.001 |
| * Refers to six-month period prior to a study visit. | | | |

In bivariable analyses conducted among the expanded study sample (*n* = 1877), individuals who reported having used a SIF every couple of weeks to every couple of days had a significantly lower hazard of dying compared to those who used SIFs once a month or less frequently (HR = 0.56; 95% CI = 0.33 – 0.96, *p* = 0.034). However, risk of all-cause mortality did not significantly differ between ≥daily SIF users and those who used SIFs once a month or less frequently in bivariable analyses (HR = 0.93; 95% CI = 0.51 – 1.70, *p* = 0.813). Table S2.1 presents the multivariable results for the association between the three-level measure of SIF use and all-cause mortality among the expanded study sample. As shown, ≥biweekly to <daily SIF use (compared to ≤once monthly or no SIF use) remained significantly associated with reduced risk of all-cause mortality after adjusting for age, sex, unstable housing, HIV serospositivity, public injection, enrolment in addiction treatment and calendar year of interview (HR = 0.49; 95% CI = 0.29 – 0.85, *p* = 0.010). However, ≥daily SIF use (compared to ≤once monthly or no SIF use) was not significantly associated with all-cause mortality after adjusting for the same set of potential confounders (HR = 0.84; 95% CI = 0.45 – 1.57, *p* = 0.590).

| **Table S1.2 Adjusted Cox regression analyses of factors associated with all-cause mortality among people who inject drugs (*n* = 1877) in Vancouver, Canada, 2006-2017.** | | | |
| --- | --- | --- | --- |
| **Variable** | **Adjusted  Hazard Ratio  (AHR)** | **95% Confidence Interval (CI)** | ***p -* value** |
| **Age** |  |  |  |
| (per year older) | 1.05 | (1.02 – 1.08) | 0.001 |
| **Sex** |  |  |  |
| (male vs. female) | 1.20 | (0.76 – 1.88) | 0.439 |
| **Unstable housing*** |  |  |  |
| (yes vs. no) | 1.77 | (1.10 – 2.83) | 0.018 |
| **HIV seropositive*** |  |  |  |
| (yes vs. no) | 4.26 | (2.88 – 6.30) | <0.001 |
| **Supervised injection facility use*** |  |  |  |
| (biweekly to every couple of days vs. ≤once a month) | 0.49 | (0.29 – 0.85) | 0.010 |
| (≥daily vs. ≤once a month) | 0.84 | (0.45 – 1.57) | 0.590 |
| **Public injection*** |  |  |  |
| (yes vs. no) | 1.45 | (0.96 – 2.18) | 0.080 |
| **Enrolled in addiction treatment*** |  |  |  |
| (yes vs. no) | 0.64 | (0.44 – 0.94) | 0.022 |
| **Calendar year of interview** |  |  |  |
| (per year increase) | 0.61 | (0.49 – 0.76) | <0.001 |
| * Refers to six-month period prior to a study visit. | | | |
