# Supplementary material for: Supervised injection facility use and all-cause mortality among people who inject drugs in Vancouver, Canada: A cohort study
Source: PLoS Med. 2019 Nov 26;16(11):e1002964. doi: 10.1371/journal.pmed.1002964 (PMC6879115; doi:10.1371/journal.pmed.1002964)
Supplement: S2 Text — ACCESS, AIDS Care Cohort to evaluate Exposure to Survival Services; VIDUS, Vancouver Injection Drug Users Study. (DOCX) [file pmed.1002964.s003.docx]

**S2 Text - Data analysis plan**

**Date of request**: June 16, 2018

**Name(s)**: Mary Clare Kennedy, Kanna Hayashi, M-J Milloy, Evan Wood & Thomas Kerr

**Cohort(s)**: ACCESS/VIDUS

**Reference Questionnaire (include name and date approved)**: ACCESS/VIDUS follow-up #17 (06/01/2014)

**Study Title**: Supervised injection facility use and all-cause mortality

Study Context (including objectives and hypotheses): Drawing on data from the VIDUS and ACCESS cohorts, we propose to examine the association between frequent supervised injection facility (SIF) use and all-cause mortality. We hypothesize the frequent SIF use will be independently and inversely associated with risk of all-cause mortality.

Study Period: VIDUS /ACCESS Baseline (BL) and Follow-up (FU) surveys (December 2006 - June 30 2017)

Study Sample (including inclusion and exclusion criteria): All VIDUS/ACCESS participants who completed at least a baseline or follow-up interview during the aforementioned study period in which they reported past-6-month injection drug use AND who reported past-6-month SIF use in ≥50% of available study visits.

Main outcome measure(s): All-cause mortality (and specific types of mortality), ascertained through linkage with the BC Vital Statistics Agency. Please use the latest update from BC Vital Statistics to ensure that data on the number of deaths and causes of death are as complete as possible.

Please classify causes of mortality into the following eight categories based on ICD-10 codes (table showing classification below):

1) HIV-related

2) Overdose

3) Liver-related

4) Homicide

5) Suicide

6) Other accidental (e.g., homicide, suicide, transport accidents, accidental falls)

7) Other non-accidental (e.g., circulatory disease, respiratory disease, neoplasms)

8) Ill-defined/unknown causes

| **Classification of causes of death by *ICD-10* code.** | | |
| --- | --- | --- |
| **Cause of death** | **ICD-10** | **Description** |
| HIV-related | B20–24 | HIV disease |
| Overdose | X40–49 | Accidental poisoning by and exposure to noxious substances |
| Liver-related | B15–19 | Viral hepatitis |
|  | B94.2 | Sequelae of viral hepatitis |
|  | C22 | Malignant neoplasm of liver and intrahepatic bile ducts |
|  | K70–77 | Diseases of liver |
| Homicide | X85–Y09 | Assault |
|  | Y87.1 | Sequelae of assault |
| Suicide | X60–X84 | Intentional self-harm |
|  | Y87.0 | Sequelae of intentional self-harm |
| Other accidental | F11–16, F19, F55 | Mental and behavioural disorders due to psychoactive substance use |
|  | V01–V99, Y85.0, Y85.9 | Transport accidents |
|  | W65–W74 | Accidental drowning |
|  | W00–W19 | Accidental falls |
|  | W24, W28–W31 | Accident caused by machinery |
|  | W32–W34 | Accidental firearm discharge |
|  | X00–X09 | Exposure to smoke, fire and flames |
|  | W20–W23, W25–W27, W35–W64, W75–W99, X10–X39, X50–X59, Y35–Y36, Y40–Y84, Y86, Y88 | All other accidents |
|  | Y10–Y34, Y87.2 | External events of undetermined intent |
|  | Y89 | Sequelae of other external causes |
|  | S00–T98 | Injury, poisoning and certain other consequences of external causes |
| Other non-accidental | All others |  |
| Ill-defined/unknown | R95–99 | Ill-defined and unspecified causes of mortality |
| *ICD-10: International Classification of Diseases, 10th edition.* | | |

**Primary statistical analyses**:

Provide frequencies (n / %) of VIDUS/ACCESS participants (who completed at least one study visit during the study period) that were excluded from the analytic sample because they: (1) did not report past-six-month injection drug use in any interviews during follow-up; or (2) did not report past-six-month SIF use in at least 50% of available study visits.

Compare the baseline characteristics (all variables listed in the table below except frequent SIF use) of participants included in the analytic sample with those excluded using the Pearson’s χ^2^ test for categorical variables and the Wilcoxon rank-sum test for continuous variables. Also provide the proportion of participants in the respective levels of each categorical variable and the median and interquartile range [IQR]) for continuous variables, stratified by being included or excluded from the study sample. Estimate unadjusted odds ratios and associated 95% confidence intervals (CIs) to compare the baseline characteristics of included versus excluded participants. Use the same methods to compare the baseline characteristics of individuals who reported past-six-month SIF use in at least 50% of their available study visits versus those who did not among cohort participants who reported past-six-month SIF use at least once during the study period.

Please provide the total person-years of observation contributed by the analytic sample. Please provide the median (and IQR) follow-up time (in months*)* for the analytic sample.

Compare the baseline characteristics of participants (for all variables listed in the table below except frequent SIF use), stratified by frequent SIF use at baseline (using definition of frequent SIF use shown in table below), by providing proportions (categorical variables) and medians/IQRs (continuous variables). Also provide unadjusted odds ratios and associated 95% CIs for these associations.

Provide frequencies of the total number of deaths and calculate a crude mortality rate (CMR) (per 1,000 person years) and a 95% CI for all-cause mortality using the Poisson distribution.

Provide frequencies for each cause of death (based on the 8 categories noted above) and calculate CMRs per 1,000 person years and 95% CIs using the Poisson distribution.

Calculate the years of potential life lost (YPLL) for each decedent using life expectancy estimates based on latest data available (from the year 2017) for the province of BC from Statistics Canada (84.6 years for females and 80.1 years for males). Calculate the median YPLL per death and rate of YPLL per 100,000 population.

Use bivariable extended Cox regression to identify factors associated with time to *all-cause mortality* (explanatory variables shown in table below). Treat all time-varying variables as time-updated.

| **Variable** | **Comparisons** |
| --- | --- |
| Age | Per year older |
| Ancestry | White vs. other |
| Sex | Male vs. female |
| Residency in Downtown Eastside* | DTES *vs. o*thers |
| Frequent SIF use* | ‘Once a week’ or greater use vs. ‘every couple of weeks’ or lower |
| Daily Heroin injection* | ≥ daily vs. < daily |
| Daily Cocaine injection* | ≥ daily vs. < daily |
| Daily Crystal methamphetamine injection* | ≥ daily vs*.* < daily |
| Daily PO injection or non-injection use* | ≥ daily vs. < daily (please include all POs as defined in previous studies) |
| Benzodiazepine use* | Yes vs. no (any use) as defined in previous studies |
| Daily Crack cocaine smoking* | ≥ daily vs. < daily |
| Daily Cannabis use* | ≥ daily vs. < daily |
| Heavy alcohol use (NIAAA-defined)* | >3 drinks in one day in past week or >7 per week (females); > 4 drinks in one day in past week or >14 per week (males) vs. Other |
| Drug injecting in public* | Yes vs. No |
| Binge injection drug use* | Yes vs. No |
| Enrollment in drug or alcohol treatment* | Yes vs. No |
| Non-fatal overdose* | Yes vs. No |
| Exposure to violence* | Yes vs. No |
| Unstable housing* | (single room occupancy hotel, shelter, recovery or transition house, jail, on the street, or having no fixed address vs. apartment or house) |
| Sex work involvement * | Yes vs. no |
| Incarceration* | Yes vs no. |
| HCV serostatus* | Positive vs. negative |
| HIV serostatus* | Positive vs. negative |
| Calendar year of interview | Per year increase |
| *Refers to 6-month period preceding a study visit | |

To estimate the independent association between frequent SIF use and all-cause mortality, first fit a multivariable model that includes frequent SIF use and all other explanatory variables shown in the table above. Next, remove the secondary explanatory variable corresponding to the smallest relative change in the frequent SIF use coefficient. Continue this iterative process until the minimum change of the value of the coefficient for frequent SIF use exceeds 5%. Lastly, force age, sex and unstable housing into the final model as explanatory variables if these are not retained in the multivariable model using the aforementioned methods.

**Sensitivity analyses (not prespecified):**

Replicate the above-detailed bivariable and multivariable extended Cox regression analyses but with an alternative three-level measure of SIF use (responses classified as the following: ≥daily vs. every couple of weeks to every couple of days vs. no use to once a month) in place of the binary measure of SIF use used in the main analysis. Follow the same multivariable model building procedure conducted for the main analysis (detailed above) to estimate the independent association between this three-level measure of SIF use and the outcome of all-cause mortality.

For the following sensitivity analyses, please apply the following study sample inclusion criteria (in place of the sample inclusion criteria used for the main analysis): All VIDUS/ACCESS participants who completed at least a baseline or follow-up interview during the study period in which they reported past-6-month injection drug use. Next, conduct bivariable and multivariable extended Cox regression analyses to examine the association between the above-mentioned three-level measure of SIF use and all-cause mortality. Follow the same procedures to build a multivariable model as those used in the main analysis.
