# Supplementary material for: Supervised injection facility use and all-cause mortality among people who inject drugs in Vancouver, Canada: A cohort study
Source: PLoS Med. 2019 Nov 26;16(11):e1002964. doi: 10.1371/journal.pmed.1002964 (PMC6879115; doi:10.1371/journal.pmed.1002964)
Supplement: S3 Text — ACCESS, AIDS Care Cohort to evaluate Exposure to Survival Services; PWID, people who inject drugs; SIF, supervised injection facility; VIDUS, Vancouver Injection Drug Users Study. (DOCX) [file pmed.1002964.s004.docx]

**S3 Text – Baseline characteristics of people who inject drugs (PWID) included vs. excluded from the main analytic sample on the basis of SIF use during follow-up**

Table S3.1 reports the baseline characteristics of VIDUS and ACCESS participants who completed at least one study visit during the study period (December 1, 2006 and June 30, 2017) in which they reported having injected drugs in the previous six months (*n* = 1877), stratified by reporting past-six-month SIF use in ≥50% of available study visits (a criterion for inclusion in the main analytic sample). As shown, compared with PWID who reported past-six-month SIF use in ≥50% of available study visits, those who did not report past-six-month SIF use in ≥50% of their study visits (and were therefore excluded from the main analytic sample) were less likely to reside in the Downtown Eastside (65.9% vs. 80.5%), be unstably housed (70.6% vs. 81.9%), inject heroin ≥daily (20.2% vs. 42.2%), inject cocaine ≥daily (7.8% vs. 12.2%), inject crystal methamphetamine ≥daily (4.1% vs. 10.3%), use prescription opioids ≥daily (6.1% vs. 10.2%), inject in public (31.4% vs 58.8%), binge inject (24.5% vs. 32.6%), have had a recent non-fatal overdose (6.9% vs 11.9%), have recently experienced violence (20.5% vs. 30.1%), and have been recently incarcerated (12.6% vs 24.5%). Additionally, PWID who did not report past-six-month SIF use in ≥50% of their study visits were more likely than those who did to be older (median age of 44 vs. 39) and be HIV seropositive (47.9% vs. 30.3%).

| **Table S3.1. Characteristics of 1877 people who inject drugs in Vancouver, Canada, stratified by reporting past-six-month supervised injection facility use in ≥50% of available study visits.** | | | | |
| --- | --- | --- | --- | --- |
| **Characteristic** | **Total (%)**  **(*n* = 1877)** | **Past-6-month SIF**^‡^ **use in ≥50% of available study visits** | | **Odds Ratio**  **(95% CI^#^)** |
|  |  | **Yes (*%*)**  **(*n* = 811)** | **No (%)**  **(*n* = 1066)** |  |
| **Age** |  |  |  |  |
| Median [IQR]^†^ | 42 [34-48] | 39 [33-46] | 44 [36-49] | 0.96 (0.95 – 0.97) |
| **Sex** |  |  |  |  |
| Male | 1224 (65.6) | 532 (65.7) | 692 (65.6) | 1.00 (0.83 – 1.22) |
| Female | 641 (34.4) | 278 (34.3) | 363 (34.4) |  |
| **Ancestry** |  |  |  |  |
| White | 1176 (63.2) | 526 (64.9) | 650 (61.8) | 1.15 (0.95 – 1.39) |
| Non-white | 686 (36.8) | 284 (35.1) | 402 (38.2) |  |
| **Downtown Eastside residence*** |  |  |  |  |
| Yes | 1355 (72.2) | 653 (80.5) | 702 (65.9) | 2.14 (1.73 – 2.66) |
| No | 522 (27.8) | 158 (19.5) | 364 (34.2) |  |
| **Unstable housing*** |  |  |  |  |
| Yes | 1410 (75.5) | 663 (81.9) | 747 (70.6) | 1.88 (1.50 – 2.35) |
| No | 458 (24.5) | 147 (18.2) | 311 (29.4) |  |
| **HIV seropositive*** |  |  |  |  |
| Yes | 757 (40.3) | 246 (30.3) | 511 (47.9) | 0.47 (0.39 – 0.57) |
| No | 1120 (59.7) | 565 (69.7) | 555 (52.1) |  |
| **Hepatitis C seropositive*** |  |  |  |  |
| Yes | 1585 (84.7) | 691 (85.3) | 894 (84.2) | 1.09 (0.85 – 1.41) |
| No | 287 (15.3) | 119 (14.7) | 168 (15.8) |  |
| **Heroin injection*** |  |  |  |  |
| ≥Daily | 557 (29.7) | 342 (42.2) | 215 (20.2) | 2.89 (2.35 – 3.54) |
| <Daily | 1320 (70.3) | 469 (57.8) | 851 (79.8) |  |
| **Cocaine injection*** |  |  |  |  |
| ≥Daily | 182 (9.7) | 99 (12.2) | 83 (7.8) | 1.65 (1.21 – 2.24) |
| <Daily | 1694 (90.3) | 711 (87.8) | 983 (92.2) |  |
| **Crystal methamphetamine injection*** |  |  |  |  |
| ≥Daily | 127 (6.8) | 83 (10.3) | 44 (4.1) | 2.66 (1.82 – 3.87) |
| <Daily | 1748 (93.2) | 726 (89.7) | 1022 (95.9) |  |
| **Non-injection crack cocaine use*** |  |  |  |  |
| ≥Daily | 689 (36.8) | 314 (38.8) | 375 (35.2) | 1.17 (0.96 – 1.41) |
| <Daily | 1186 (63.3) | 496 (61.2) | 690 (64.8) |  |
| **Prescription opioid use*** |  |  |  |  |
| ≥Daily | 148 (7.9) | 83 (10.2) | 65 (6.1) | 1.75 (1.25 – 2.46) |
| <Daily | 1728 (92.1) | 728 (89.8) | 1000 (93.9) |  |
| **Cannabis use*** |  |  |  |  |
| ≥Daily | 432 (23.0) | 174 (21.5) | 258 (24.2) | 0.86 (0.69 – 1.07) |
| <Daily | 1443 (77.0) | 635 (78.5) | 808 (75.8) |  |
| **Benzodiazepine use*** |  |  |  |  |
| Yes | 71 (3.8) | 28 (3.5) | 43 (4.0) | 0.85 (0.52 – 1.38) |
| No | 1806 (96.2) | 783 (96.6) | 1023 (96.0) |  |
| **Heavy alcohol use*^** |  |  |  |  |
| Yes | 244 (13.0) | 96 (11.8) | 148 (13.9) | 0.83 (0.63 – 1.09) |
| No | 1631 (87.0) | 715 (88.2) | 916 (86.1) |  |
| **Public injection*** |  |  |  |  |
| Yes | 808 (43.3) | 476 (58.8) | 332 (31.4) | 3.12 (2.58 – 3.77) |
| No | 1057 (56.7) | 333 (41.2) | 724 (68.6) |  |
| **Binge injection*** |  |  |  |  |
| Yes | 522 (28.0) | 264 (32.6) | 258 (24.5) | 1.49 (1.22 – 1.83) |
| No | 1340 (72.0) | 545 (67.4) | 795 (75.5) |  |
| **Non-fatal overdose*** |  |  |  |  |
| Yes | 169 (9.0) | 96 (11.9) | 73 (6.9) | 1.83 (1.33 – 2.51) |
| No | 1706 (91.0) | 714 (88.2) | 992 (93.2) |  |
| **Enrolled in addiction treatment*** |  |  |  |  |
| Yes | 1010 (54.0) | 426 (52.6) | 584 (55.0) | 0.91 (0.76 – 1.09) |
| No | 862 (46.0) | 384 (47.4) | 478 (45.0) |  |
| **Exposure to violence*** |  |  |  |  |
| Yes | 459 (24.6) | 242 (30.1) | 217 (20.5) | 1.67 (1.35 – 2.07) |
| No | 1407 (75.4) | 563 (69.9) | 844 (79.6) |  |
| **Sex work involvement*** |  |  |  |  |
| Yes | 320 (17.1) | 148 (18.3) | 172 (16.2) | 1.17 (0.92 – 1.49) |
| No | 1552 (82.9) | 659 (81.7) | 893 (83.9) |  |
| **Incarceration*** |  |  |  |  |
| Yes | 332 (17.7) | 198 (24.5) | 134 (12.6) | 2.26 (1.77 – 2.87) |
| No | 1541 (82.3) | 610 (75.5) | 931 (87.4) |  |
| Note: Column counts may not necessarily sum to column totals due to missing baseline data and column percentages may not necessarily sum to 100% due to rounding error.  ^‡^ SIF = supervised injection facility.  *Refers to the 6 months prior to a baseline interview.  ^†^ IQR = interquartile range.  ^#^ CI = confidence interval.  ^Average of >3 alcoholic drinks on at least 1 day per week or >7 drinks in total per week (women), or >4 alcoholic drinks on at least 1 day per week or >14 drinks in total per week (men). | | | | |
